# Transcriptome and phytohormone analysis reveal mechanism of gall formation by *Trichagalma acutissimae* larvae on oak leaves

**DOI:** 10.3389/fpls.2025.1646230

**Published:** 2025-09-18

**Authors:** Yingnan Wang, Chao Xue, Saisai Wu, Yuanchen Zhang, Ran Li, Yujian Li, Xianfeng Yi

**Affiliations:** ^1^ School of Life Sciences, Qufu Normal University, Qufu, China; ^2^ College of Biological and Food Engineering, Anyang Institute of Technology, Anyang, China

**Keywords:** *Trichagalma acutissimae*, gall induction, transcriptome, phytohormone, oak

## Abstract

**Introduction:**

Gall formation caused by plant-organism interactions affects plant development and is essential for the life cycle of gall-inducing insects. Plant hormones like auxins and cytokinins, regulate gall development and defense responses. Despite the extensive morphological characterization of galls, the molecular mechanisms underlying gall induction remain largely unresolved.

**Methods:**

In this study, we quantified hormone concentrations and performed transcriptome analyses to investigate the mechanisms by which leaf galls are induced by the cynipid wasp *Trichagalma acutissimae* on two oak host species, *Quercus variabilis* and *Q. acutissima*.

**Results:**

Our preliminary results indicate that wasp larvae may synthesize auxins and cytokinins—a conclusion supported by the gall transcriptome data. Downregulation of IAA biosynthesis genes in gall tissues coincides with significantly higher IAA levels in the larvae compared to the leaves and galls. Likewise, the detection of active cytokinins in the larvae indicates their ability to synthesize cytokinins autonomously. Furthermore, we observed significant suppression of jasmonic acid (JA) biosynthesis in the gall tissues, which strongly supports the nutritional hypothesis. We also identified the upregulation of biosynthetic genes involved in carbohydrate metabolism, amino acid metabolism, and lipid metabolism, providing evidence for the ‘nutritional hypothesis’ of gall formation.

**Discussion:**

This integrative exploration of hormonal dynamics and transcriptomic changes offers insights into the mechanisms of gall induction.

## 1 Introduction

Gall formation, characterized by localized and abnormal tissue proliferation induced by gall-inducing organisms such as insects, fungi, or bacteria, represents a distinctive example of plant-parasite interaction (Wang et al., 2025). Galls formed by parasitic insects provide specialized microhabitats, facilitating insect development and reproduction while simultaneously influencing the growth, physiology, and defense responses of the host plants (Santos et al., 2011; Tooker and Helms, 2014; Zhang et al., 2015; Desnitskiy et al., 2023). These interactions significantly affect the host plants by redirecting nutrients and modifying plant metabolism, creating a protected niche that supplies abundant resources essential for the survival and growth of the inducing insects (Dorchin et al., 2006; Kutsukake et al., 2019; Harris and Pitzschke, 2020). Therefore, understanding the mechanisms underlying gall formation is of great importance in agricultural and forestry contexts, as galls can negatively impact crop yields and pose challenges for pest management strategies (Pujade and Wang, 2012). Although extensive morphological and ecological descriptions of galls have been documented (Ferreira et al., 2019; Rezende et al., 2021), the detailed molecular mechanisms that govern gall induction and development remain insufficiently understood, primarily due to the complex and species-specific interactions between gall-inducing organisms and their host plants (Yamaguchi et al., 2012; Bartlett and Connor, 2014; Li et al., 2017; Body et al., 2019; Roy and Das, 2023).

Previous research has indicated that gall formation involves intricate interactions among insect-derived chemical signals, plant hormones (e.g., auxins, cytokinins, and abscisic acid), and specific regulatory proteins. Plant hormones are critical in controlling cell proliferation, differentiation, and stress responses during gall formation (Davies, 1995; Tanaka et al., 2013; Acevedo et al., 2019). Numerous studies have demonstrated that the levels of plant hormones fluctuate significantly upon gall infestation, exhibiting clear differences between gall tissues and non-infested plant tissues (Jia et al., 2020; Wang et al., 2022). For example, elevated salicylic acid levels observed in gall tissues highlight the role of plant hormones not only in gall development but also in mediating host defense mechanisms (Lieceng et al., 2011). Despite extensive documentation of hormone level variations between galled and healthy tissues, the precise molecular regulatory pathways driving these hormonal changes are still largely unknown.

Advancements in transcriptomic technologies have opened new opportunities for exploring the molecular basis of gall formation, substantially improving our understanding of plant-insect interactions. Transcriptome analyses have successfully identified key genes associated with gall development, plant defensive responses, and hormone-related signaling pathways, offering deeper insights into the genetic and biochemical bases of host-parasite interactions (Dong and Chen, 2013). For instance, Arabidopsis response regulator 5 (ARR5), a primary cytokinin-responsive gene, has been found significantly upregulated in galls induced on various plant species, mediating peptide signaling related to cell division and altering hormonal sensitivity (Shi et al., 2019). Similarly, transcriptome studies of psyllid-induced galls on Hawaiian *Metrosideros* species identified multiple auxin-responsive genes associated with auxin and brassinolide signaling pathways (Bailey et al., 2015).

The ecological interactions among plants, gall-inducing insects, and galls are particularly intimate and sophisticated. Gall induction generally imposes moderate stress on host plants but rarely results in severe damage or mortality. This interaction typically confines significant physiological changes to the localized gall-forming sites, while the overall metabolism of the host plant remains relatively stable (Raman et al., 2006). Gall-inducing insects have evolved refined adaptations enabling them to manipulate host plant physiology more effectively than their non-galling counterparts (Raman et al., 2005; Schaefer et al., 2005; Shorthouse et al., 2005; Espírito-Santo and Fernandez, 2007). This plant-insect interaction carries profound ecological and evolutionary implications, shaping plant-insect dynamics and significantly contributing to the biodiversity of oak forest ecosystems. Therefore, clarifying the mechanisms underlying gall induction not only enriches theoretical insights into host-parasite coevolutionary processes but also informs practical pest management strategies.

**Figure 1:**
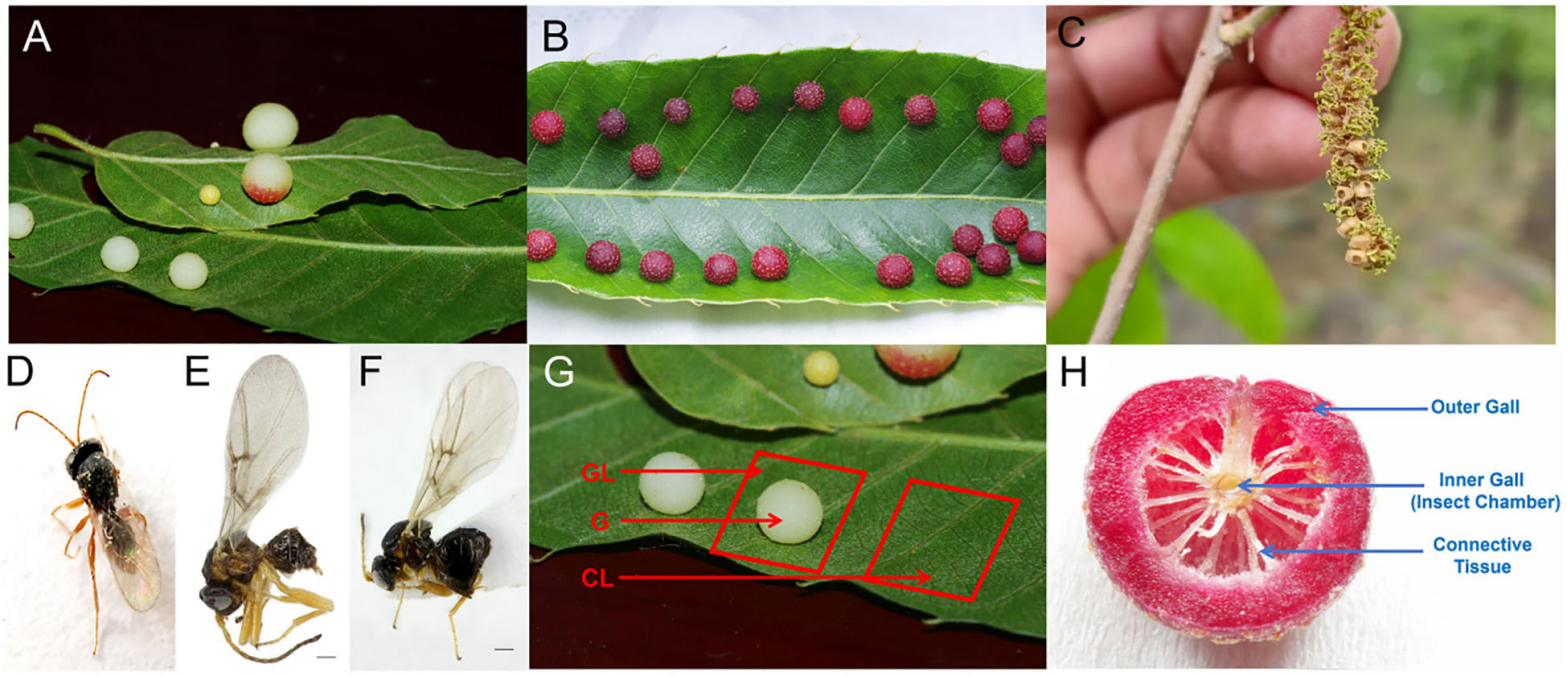
Galls induced by *Trichagalga acutissimae* on the leaves and catkins of *Quercus variabilis* and *Q. acutissima*. **(A)** Asexual galls on *Q. acutissima* leaves; **(B)** Asexual galls on *Q. variabilis* leaves; **(C)** Sexual galls on catkins; **(D)** Asexual adult; **(E)** Sexual male; **(F)** Sexual female; **(G)** Schematic representation of sample types; **(H)** Illustration of the internal structure of the gall.

*Trichagalma acutissimae* is a major pest affecting oak species (Wang et al., 2017; Xue et al., 2020; Wang et al., 2025), particularly *Quercus variabilis* and *Q. acutissima* in China (Melika et al., 2010). *T. acutissimae* exhibits both sexual and asexual generations and induces galls on the leaves and catkins of *Q. variabilis* and *Q. acutissima* (**Figures 1A–C**), affecting reproduction, growth, and development of hosts. Adults of asexual generation (**Figure 1D**) emerge in autumn and oviposit in the dormant buds, leading to sexual gall formation on the catkins in the next spring (Wang J. et al., 2016). Then, the sexually reproducing males and females emerge from the sexual galls (**Figures 1E, F**), following copulation between April and May, and oviposit on the leaves of oak trees. This process induces the formation of round, succulent asexual galls on either the front or back of leaf veins, depending on the host species (**Figures 1A, B**). Even though the life history of *T. acutissimae* is relatively clear, the mechanism by which succulent asexual galls are induced remains largely unknown. This study employs comprehensive transcriptomic analyses of gall tissues and adjacent host tissues from these two oak species. First, we quantified hormone concentrations in host leaves, gall shells, and gall-inhabiting larvae to explore hormonal roles in gall initiation and development. Second, we performed transcriptome sequencing across different tissue types (asexual galls, galled leaves, and healthy leaves) to identify candidate genes involved in gall developmental processes. By integrating hormone level measurements with transcriptomic data, we tested two hypotheses regarding the influence of hormonal differences, gene expression variations, and functional gene alterations on gall induction: (1) The production of phytohormones by larvae plays a critical role in promoting gall formation, and (2) the suppression of host defense mechanisms and the reprogramming of nutrient allocation pathways contribute to facilitating insect development. The outcomes of this research will elucidate the complex regulatory networks involved in gall formation, thereby significantly advancing our understanding of plant-insect interactions. Additionally, the findings will offer essential insights for the development of effective pest management strategies.

## 2 Materials and methods

### 2.1 Sample collection

Healthy and galled leaves were collected in June 2023 from five mature individuals of each oak species, *Q. variabilis* and *Q. acutissima*, in Shimenshan Town, Qufu City, Shandong Province, China. From the galled leaves, an approximately 1 cm × 1 cm tissue sample was excised directly beneath the asexual galls (**Figure 1G**). During gall sampling, we selected galls of comparable developmental stage, size, and color from both *Q. variabilis* and *Q. acutissima*. Control samples of identical size were collected from the corresponding anatomical position on opposite sides of healthy leaves from the same branch. Galls were subsequently dissected with sterilized scalpels and tweezers to separate gall shells from larvae (**Figure 1H**). Each tissue type included five biological replicates for each oak species. All samples were immediately frozen in liquid nitrogen before use. Samples collected from *Q. variabilis* were divided into two groups for hormone analysis and transcriptome sequencing, respectively, whereas samples from *Q. acutissima* were used solely for hormone quantification. In this study, only *Q. variabilis* was selected for transcriptome analysis due to two facts: *Q. variabilis* is dominant in this area and *T. acutissimae* often parasitizes *Q. variabilis* rather than *Q. acutissima*.

### 2.2 Determination of plant hormones

Plant hormone quantification was performed as follows: Samples were ground thoroughly in liquid nitrogen and accurately weighed into test tubes (100 mg FW). A 10 mL acetonitrile extraction solution, mixed with 8 μL stock solution containing the following internal standards (all at 50 μg/mL concentration): [²H_5_]-indole-3-acetic acid ([²H_5_]-IAA), [²H_5_]-jasmonic acid ([²H_5_]-JA), [²H_6_]-zeatin ([²H_6_]-Z), [²H_5_]-trans-zeatin riboside ([²H_5_]-TZR), [²H_6_]-isopentenyl adenine ([²H_6_]-IP), and [²H_6_]-isopentenyl adenosine ([²H_6_]-IPA), with all internal standards having purity >98% (Cambridge Isotope Laboratories, USA) was added, and samples were extracted overnight at 4 °C. Extracts were centrifuged at 12,000 g for 5 min at 4 °C, and the supernatants were collected. Pellets were re-extracted twice, each time using 5 mL acetonitrile solution, and supernatants were combined. Impurities were purified using appropriate amounts of C18 and GCB (every 10 mL of supernatant, 0.5–1 g of C18 was added), followed by centrifugation at 12,000 g for 5 min at 4 °C. The purified extracts were dried under nitrogen gas, resuspended in 400 μL methanol, filtered through a 0.22 μm organic-phase membrane, and stored at -20 °C before analysis. Hormone measurements, including indole-3-acetic acid (IAA), jasmonic acid (JA), zeatin, trans-zeatin riboside (TZR), isopentenyl adenine (IP), and isopentenyl adenosine (IPA), were conducted using an Agilent 1290 high-performance liquid chromatography (HPLC) coupled with an AB Qtrap 6500 mass spectrometer. Internal standards were included for accurate quantification. Hormone detection was entrusted to Nanjing Ruiyuan Biotechnology Co., Ltd.

### 2.3 RNA extraction, library construction, and sequencing

Total RNA was extracted from 15 samples, including five replicates each of gall (G, larvae excluded), galled leaves (GL), and control leaves (CL) using TRIzol reagent. RNA purity and concentration were assessed using a NanoDrop 2000 spectrophotometer (Thermo Scientific, USA), while RNA integrity was verified using an Agilent 2100 Bioanalyzer (Agilent Technologies, CA, USA). Transcriptome libraries were constructed utilizing the VAHTS Universal V6 RNA-seq Library Prep Kit. Libraries underwent quality control with an Agilent 2100 Bioanalyzer, and qualified libraries were subsequently sequenced on an Illumina NovaSeq 6000 platform to generate 150 bp paired-end reads.

### 2.4 Transcriptome and differential expression analysis

The transcriptomic unigenes were obtained through *de novo* assembly. Unigeneswere functionally annotated against the NR, Swiss-Prot, KEGG, KOG, eggNOG, GO, and Pfam databases using the DIAMOND software (E-value < 1e-5) (Buchfink et al., 2015). Transcript abundance (FPKM) was quantified using bowtie2 alignment and eXpress software (Langmead and Salzberg, 2012; Roberts and Pachter, 2013). Differentially expressed genes (DEGs) were identified with DESeq2 (q-value < 0.05, fold-change > 2) (Roberts et al., 2011). Functional enrichment of DEGs was performed via GO and KEGG analyses using R software, with P-values adjusted for multiple testing correction, and results were visualized through bar plots and enrichment diagrams.

## 3 Results

### 3.1 Determination of plant hormones

**Figure 2:**
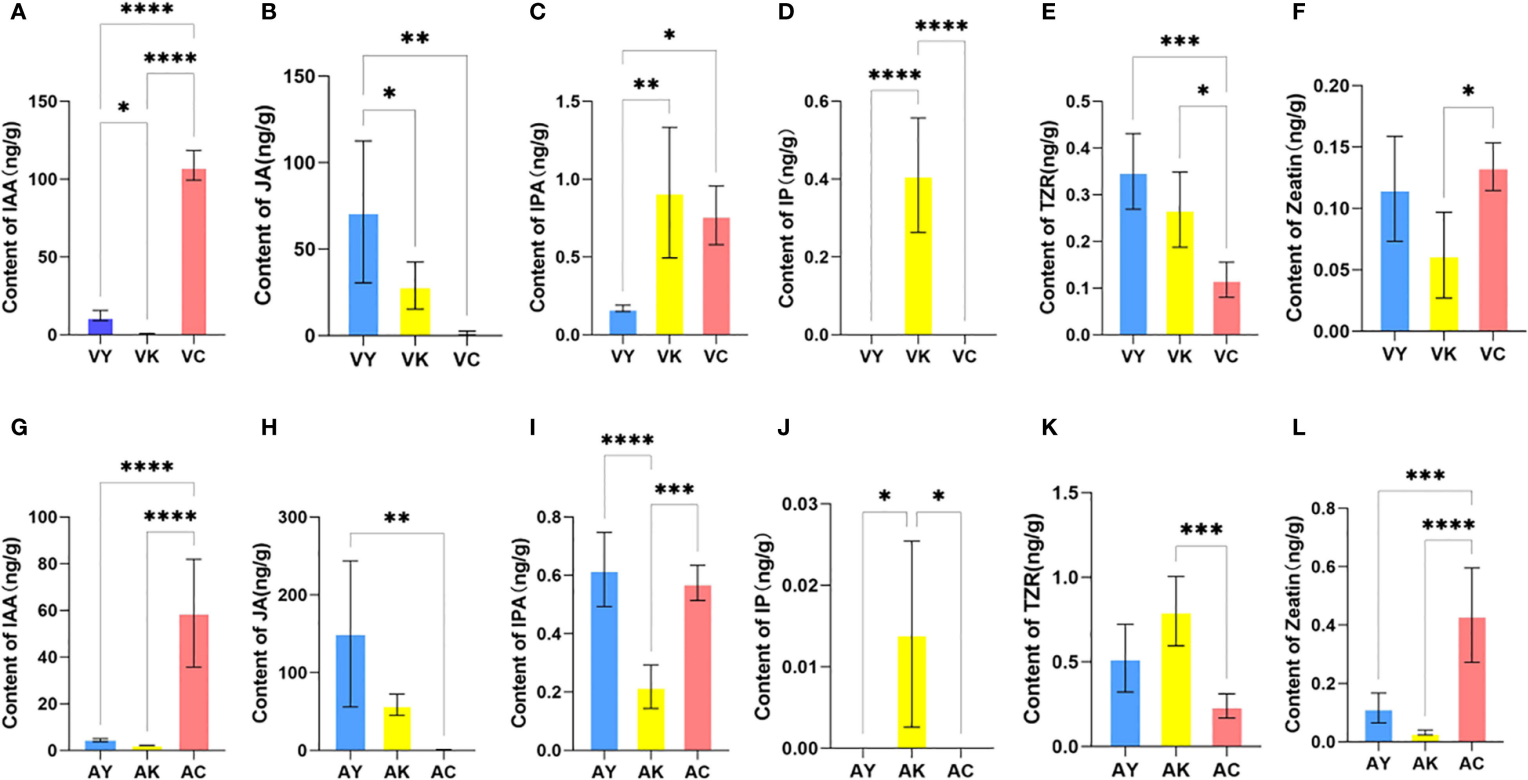
Phytohormone content of host leaves of *Quercus variabilis* (VY) and *Q. acutissima* (AY), galls on *Q. variabilis* (VK) and *Q. acutissima* (AK), and *Trichagalma acutissimae* larvae parasitized on *Q. variabilis* (VC) and *Q. acutissima* (AC). **(A, G)** IAA; **(B, H)** JA; **(C, I)** IPA; **(D, J)** IP; **(E, K)** TZR; **(F, L)** Zeatin. Data are expressed as mean ± SD. *, **, ***, and **** represent statistically significant at P < 0.05, 0.01, 0.001, and 0.0001, respectively.

Indole-3-acetic acid (IAA) concentrations differed among leaves of *Q. variabilis* (VY), galls (VK), and *T. acutissimae* larvae (VC) (**Figure 2A**; F = 517.8, df = 2, P < 0.0001). Moreover, IAA levels were significantly higher in VC compared to VY and VK (P < 0.0001, P = 0.047, respectively). Additionally, VY exhibited significantly higher IAA concentrations than VK (P = 0.002). Significant differences in jasmonic acid (JA) levels were observed among the groups (**Figure 2B**; F = 9.986, df = 2, P = 0.0028). VY exhibited the highest JA concentration, which was greater than both VC and VK (P = 0.002, P = 0.047, respectively). IPA levels varied among the samples (**Figure 2C**; F = 11.01, df = 2, P = 0.0019). The highest IPA content was detected in VK, showing an elevation over VY (P = 0.002). IPA levels in VC were also higher than those in VY (P = 0.01). Isopentenyl adenine (IP) was exclusively detected in VK (**Figure 2D**; F = 39.09, df = 2, P < 0.0001), with concentrations exceeding both VY and VC (all P < 0.0001). Trans-zeatin riboside (TZR) distribution showed variation (**Figure 2E**; F = 14.33, df = 2, P = 0.0007). Peak TZR levels occurred in VY, exceeding VC (P = 0.0005). VK displayed intermediate values higher than VC (P = 0.01). Zeatin content also differed among the samples (**Figure 2F**; F = 6.140, df = 2, P = 0.0146), with concentrations in VC being elevated compared to VK (P = 0.01).

Similarly, IAA levels differed among leaves of *Q. acutissima* (AY), galls (AK), and *T. acutissimae* larvae (AC) (**Figure 2G**; F = 28.88, df = 2, P < 0.0001). AC exhibited higher IAA concentrations compared to AY and AK (all P < 0.0001), while no difference was observed between AY and AK (P = 0.96). We showed significant variation in JA concentrations among the groups (**Figure 2H**; F = 9.373, df = 2, P = 0.0035). AY contained the highest JA levels, which were greater than those in AC (P = 0.003). We also detected differences in IPA content among the groups (**Figure 2I**; F = 28.76, df = 2, P < 0.0001). Similar IPA levels observed in AY and AC (P = 0.71), both were higher than those in AK (all P < 0.0001). IP was exclusively detected in AK (**Figure 2J**; F = 7.538, df = 2, P = 0.0076), with concentrations higher than those in AY and AC (all P = 0.02). TZR levels varied among the samples (**Figure 2K**; F = 13.55, df = 2, P = 0.0008). VK contained the highest TZR concentrations, greater than those in AC (P = 0.0006). We also observed differences in zeatin content among the groups (**Figure 2L**; F = 23.52, df = 2, P < 0.0001). The highest zeatin levels occurred in AC compared to both AY and AK (P = 0.0007; P < 0.0001).

### 3.2 Transcriptome sequencing and differential expression analysis

**Figure 3:**
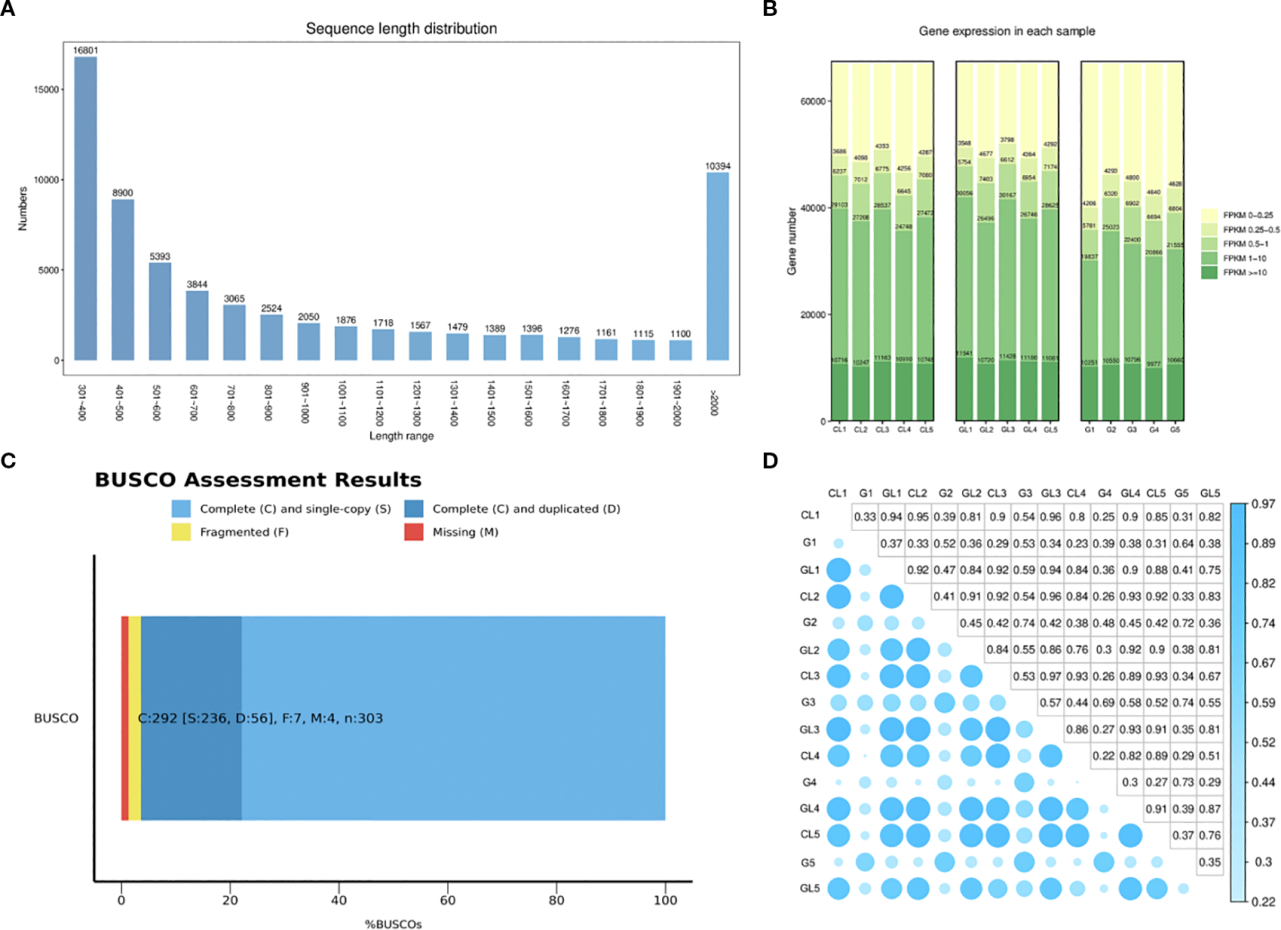
Gene expression levels in galls (G), galled leaves (GL), and control leaves (CL): **(A)** Length distribution plot of Unigenes; **(B)** FPKM-based expression distribution map; **(C)** Evaluation of transcriptome assembly quality using BUSCO; **(D)** Heatmap of correlation coefficients between samples.

In this study, a total of 15 RNA-seq libraries were constructed and sequenced, yielding approximately 105.94 gigabases (Gb) of clean, high-quality sequencing data. Each sample provided between 6.72 and 7.42 Gb of effective data, exhibiting excellent quality with Q30 percentages ranging from 93.63% to 94.43% and an average GC content of 43.69% (**Supplementary Table S1**). Following stringent quality control and filtering procedures, a total of 67,048 unigenes were successfully assembled, covering a cumulative length of approximately 73,266,917 base pairs (bp). The average unigene length was calculated at 1,092.75 bp. Length distribution analysis showed a bimodal pattern, with a significant proportion of unigenes in the length intervals of 301–400 bp and over 2000 bp, while the frequency of unigenes progressively decreased between these two intervals (**Figure 3A**). Quantitative analysis based on FPKM values revealed that over 20% of genes in all three tissue types (G/GL/CL) showed high expression levels (FPKM >10). Functional annotation against multiple databases (NR, KEGG, Swiss-Prot, Pfam, and GO) identified numerous functional genes associated with plant cell wall formation, membrane components, carbohydrate metabolism, energy metabolism, translation, amino acid metabolism, and lipid metabolism in leaves following feeding by the oak gall wasp larvae (**Figure 3B**).

BUSCO analysis revealed that over 90% of conserved orthologous genes found in closely related species were successfully recovered, with minimal gene absence, thereby indicating robust transcriptome assembly quality (**Figure 3C**). Additionally, pairwise correlation analysis of the gene expression profiles among replicates within each tissue group indicated a high degree of reproducibility and consistency (**Figure 3D**). These results collectively validated the reliability and accuracy of subsequent differential expression analyses.

Functional annotation efforts were conducted comprehensively against several widely-used databases. A significant proportion of unigenes, totaling 40,734 (60.75%), were successfully annotated against the NCBI NR database. Additionally, 27,733 (41.36%) unigenes were annotated to the Swiss-Prot database, 8,268 (12.33%) to KEGG, 21,734 (32.42%) to KOG, 35,050 (52.28%) to eggNOG, 23,186 (34.58%) to GO, and 24,482 (36.51%) to Pfam (**Table 1**). These extensive functional annotations facilitated the identification and interpretation of differentially expressed genes (DEGs) and provided a solid foundation for investigating molecular pathways potentially involved in gall formation.

**Table 1:** Summary of annotation result.

Databases	Number of unigenes	Percentage (%)
NR	40,734	60.75
Swiss-Prot	27,733	41.36
KEGG	8,268	12.33
KOG	21,734	32.42
eggNOG	35,050	52.28
GO	23,186	34.58
Pfam	24,482	36.51

**Figure 4:**
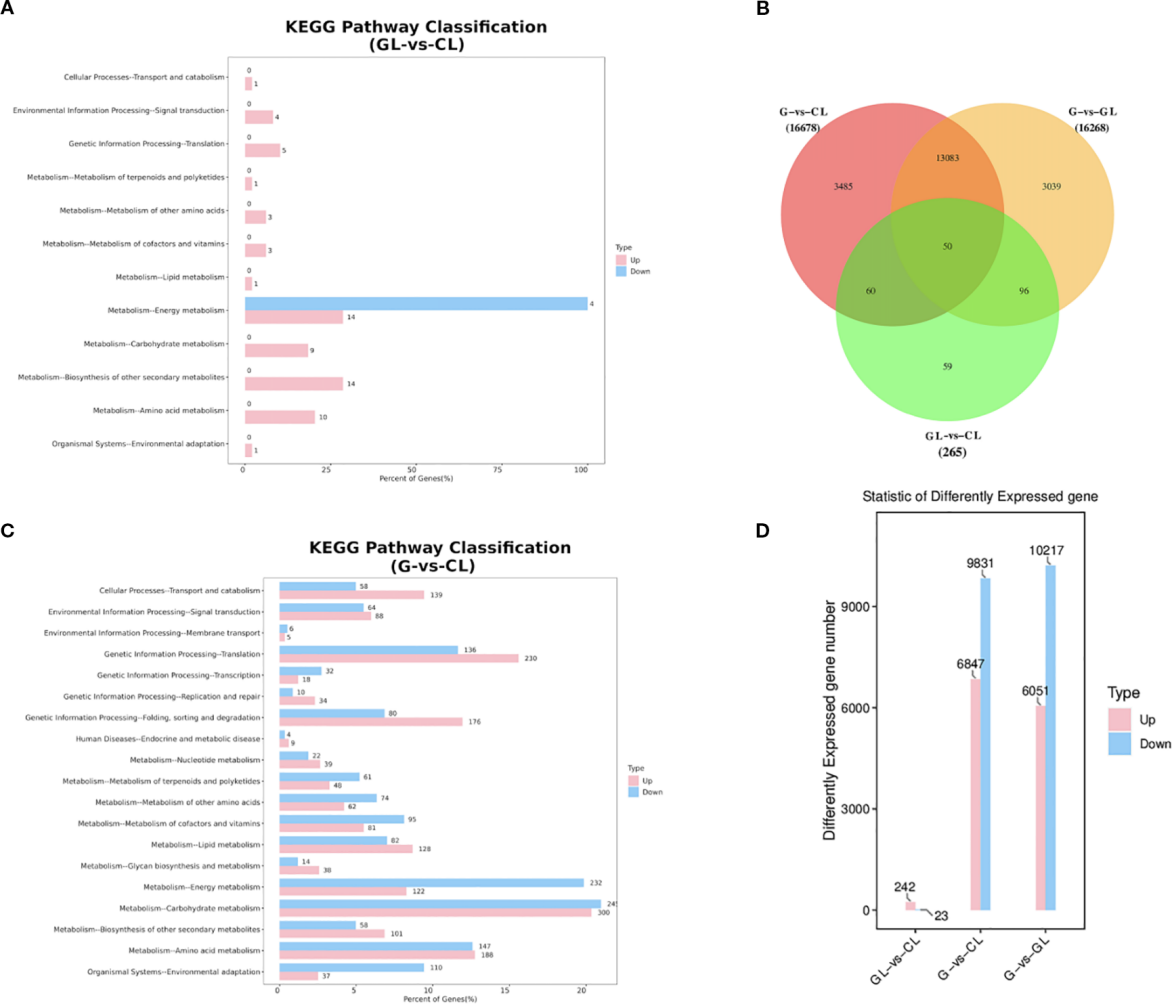
Differential gene expression analysis in GL *vs* CL, G *vs* CL, and G *vs* GL: **(A)** Comparison of the distribution of DEGs at KEGG Level 2 (GL vs CL); **(B)** Venn diagram illustrating the number of differentially expressed genes in each comparison group; **(C)** Comparison of the distribution of DEGs at KEGG Level 2 (GL vs CL); **(D)** Statistical summary of the number of differentially expressed genes in each comparison group. G, GL, and CL represent galls, galled leaves, and control leaves of *Quercus variabilis*, respectively.

Comparative analyses of gene expression across the three tissue types (GL vs CL, G vs CL, and G vs GL) identified substantial transcriptomic alterations associated with gall induction. The comparison between galled leaves and control leaves (GL vs CL) revealed 265 DEGs, of which 242 genes were upregulated and 23 were downregulated. In stark contrast, comparisons involving gall shells (G vs CL and G vs GL) exhibited dramatically higher numbers of DEGs. Specifically, 16,678 DEGs were identified in the comparison of gall shells versus control leaves (G vs CL), comprising 6,847 upregulated and 9,831 downregulated genes. Similarly, the comparison between gall shells and galled leaves (G vs GL) identified 16,268 DEGs, including 6,051 upregulated and 10,217 downregulated genes (**Figure 4D**). Venn diagram analyses highlighted both distinctiveness and minimal overlap among the three comparisons, with only 50 DEGs (0.15% of total identified DEGs) shared across GL vs CL, G vs CL, and G vs GL comparisons (**Figure 4B**). Among the unique DEGs, the comparisons of G vs CL and G vs GL yielded the most substantial numbers, identifying 3,485 and 3,039 unique DEGs, respectively. The GL vs CL comparison produced a significantly smaller set of unique DEGs (59 genes). The number of DEGs identified in G vs CL and G vs GL comparisons far exceeded that in GL vs CL, indicating substantial transcriptomic reprogramming in gall tissues compared to plant tissues.

KEGG enrichment analysis further illustrated functional trends among identified DEGs. In the GL vs CL comparison, DEGs were primarily enriched in pathways related to energy metabolism, biosynthesis of secondary metabolites, and amino acid metabolism. Conversely, DEGs in comparisons involving gall shells (G vs CL and G vs GL) showed consistent enrichment patterns across multiple metabolic and cellular processes, prominently featuring carbohydrate metabolism, energy metabolism, translation, amino acid metabolism, protein folding, sorting and degradation, and lipid metabolism pathways (**Figures 4A, C**). These metabolic alterations are closely associated with the vigorous biosynthetic activities and elevated energy demands during gall development, particularly the pronounced changes in carbohydrate and amino acid metabolism that may directly respond to the nutritional requirements of gall wasp larvae.

### 3.3 Differentially expressed genes related to plant hormone biosynthesis and signal transduction

Differentially expressed genes (DEGs) involved in plant hormone biosynthesis and signaling pathways were extensively analyzed. In the comparisons between gall shells and both leaf tissues (G vs GL and G vs CL), a total of four DEGs related to auxin (IAA) synthesis were identified. Among these genes, three were significantly down-regulated, encoding enzymes such as tryptophan aminotransferase and flavin monooxygenase, both critical for auxin biosynthesis. The downregulation of genes observed in this study, combined with elevated IAA levels in larvae, suggests a potential insect-mediated contribution to gall formation. Conversely, one flavin monooxygenase gene was notably up-regulated (**Supplementary Table S2**). Additionally, nine DEGs associated with auxin inactivation pathways were detected in these comparisons. Specifically, one ILR family gene was down-regulated, while eight genes involved in IAA degradation were up-regulated, including one DAO gene, five ILR family genes, and two GH3 family genes (**Supplementary Table S3**).

Cytokinin-related pathways also exhibited significant changes. Five DEGs involved in cytokinin synthesis were identified and mapped to the zeatin biosynthesis pathway (KEGG ko00908) (**Supplementary Table S4**). Notably, two key adenosine 5’-isoamyl phosphate transferase (IPT) genes, essential rate-limiting enzymes in the initial step of cytokinin biosynthesis, were markedly down-regulated. Additionally, one zeatin O-glucosyltransferase gene involved in cis-zeatin synthesis exhibited significant up-regulation exclusively in the comparison of gall shells versus control leaves (G vs CL). In contrast, one UDP-glycosyltransferase gene involved in dihydro-zeatin synthesis and one cytokinin dehydrogenase (CKX) gene related to cytokinin degradation were both down-regulated. Furthermore, five DEGs involved in cytokinin signaling through the two-component system were identified, including one down-regulated AHP gene and four response regulator (RR) genes. Among these RRs, the up-regulation of ARR17 (type-A RR) suggests cytokinin accumulation, whereas ARR2, ARR14, and ORR26 (all type-B RRs) were down-regulated (**Supplementary Table S4**).

Eight DEGs related to jasmonic acid (JA) biosynthesis pathways were identified. Among these genes, QuJA1, encoding an OPR-family enzyme, was significantly up-regulated in gall shells relative to control leaves (G vs CL), although no significant change was observed in gall shells relative to galled leaves (G vs GL) (**Supplementary Table S5**).

### 3.4 Differentially expressed genes related to primary metabolism

DEGs involved in primary metabolic pathways showed significant transcriptional shifts associated with gall formation. Within starch metabolism, four DEGs associated with starch synthesis were identified. Three genes encoding starch biosynthetic enzymes were up-regulated, whereas one was down-regulated (**Supplementary Table S6**). Furthermore, 33 DEGs were identified in the starch and sucrose metabolism pathway (ko00500) involved in soluble sugar metabolism. Among these genes, 25 significantly upregulated DEGs were responsible for sucrose synthase, fructokinase, hexokinase (HK), and 4-alpha-glucanotransferase genes. Conversely, eight genes, predominantly encoding beta-glucosidase (β-Glu) enzymes involved in sugar hydrolysis, were down-regulated (**Supplementary Table S7**).

Lipid metabolism also showed notable differences, with a total of 16 fatty acid-related DEGs identified. Among these, acetyl-CoA carboxylase (ACCase) was prominently represented. In total, 14 fatty acid-related DEGs exhibited up-regulation, whereas two genes were down-regulated (**Supplementary Table S8**).

Finally, amino acid metabolic pathways displayed substantial changes. A total of 18 DEGs associated with amino acid metabolism were identified, primarily encoding amino acid transporter proteins crucial for amino acid uptake and distribution. Specifically, 11 DEGs were detected in the gall shell versus control leaf comparison (G vs CL), including seven up-regulated and four down-regulated genes. Similarly, the gall shell versus galled leaf comparison (G vs GL) identified 18 DEGs, with 14 genes up-regulated and four down-regulated (**Supplementary Table S9**).

## 4 Discussion

### 4.1 Phytohormonal patterns in galls, wasps, and host plants

The initiation and development of plant galls, abnormal growths induced by various organisms, are intricately linked to plant hormones, particularly auxins (Kmieć, 2025). Numerous studies have robustly confirmed that IAA, the predominant natural auxin, plays a pivotal, key role in the earliest phases of gall formation and subsequent morphogenesis (Tooker and Helms, 2014). This study contributes significantly to this understanding by quantifying IAA distribution within the specific system of oak galls induced by the parasitic wasp *T. acutissimae*. Our results reveal a striking pattern: the larvae residing within the galls contained significantly higher concentrations of IAA compared to both the surrounding host plant tissues and the gall structure itself. Furthermore, the gall shells exhibited the lowest IAA levels detected, a finding consistent with prior previous reports in other galling systems (Yamaguchi et al., 2012). However, despite this clear association with initiation, little compelling evidence currently supports a direct, sustained involvement of IAA in the later, expansive growth phase of these oak galls specifically induced by cynipid wasps (Bedetti et al., 2014; Bedetti et al., 2017; Bedetti et al., 2018; Martinson et al., 2022). The high IAA detected within the larvae aligns with previous studies that have consistently detected IAA in various gall-inducing insects, often finding particularly high concentrations localized within their salivary glands (Suzuki et al., 2014; Ponce et al., 2021). This endogenous production capability strongly implies that these insects are not merely sequestering plant-derived IAA, but are active producers. Furthermore, it is hypothesized that they actively may transport their self-synthesized IAA into host plant tissues via saliva secreted during feeding or oviposition, thereby directly manipulating host cell division and differentiation to initiate the gall (Acevedo et al., 2019), Notably, this phytohormone production serves dual physiological functions: initiating gall formation during early infestation stages and subsequently sustaining gall development throughout larval maturation. This biphasic regulatory mechanism is substantiated by the persistent IAA gradient observed between larval tissues and developing galls, as evidenced by the maintained concentration differential. This pattern aligns with reported models in other galling systems where continuous phytohormone supply is required for gall morphogenesis (Bartlett and Connor, 2014; Tooker and Helms, 2014). The difference in IAA levels observed in this study, with larvae acting as a high-concentration source and the gall shells showing minimal amounts, provides strong corroborative evidence that a similar mechanism of insect-derived auxin production and secretion is likely adopted by *T. acutissimae* larvae. Based on these convergent findings, we hypothesize that *T. acutissimae* larvae dominate auxin secretion within the gall microenvironment and may be directly involved in IAA synthesis. While direct evidence for *de novo* IAA synthesis by the larvae remains largely lacking (Yamaguchi et al., 2012), this larval-centric hormonal activity is therefore posited to exert major control over the fundamental processes of gall formation and its initial expansion on the oak host, positioning the insect larva as the primary architect directing the plant’s developmental reprogramming through auxin manipulation (Tooker and De Moraes, 2011; Yamaguchi et al., 2012).

The potent influence of cytokinins on plant morphology is particularly evident in gall formation. Experimental research has demonstrated that the exogenous application of cytokinin-auxin mixtures can artificially induce the development of gall-like structures in plant tissues (Bartlett and Connor, 2014), underscoring the synergistic role these hormones play in triggering abnormal growth. Consequently, due to its direct impact on cell proliferation and its proven ability to initiate gall-like growth when manipulated externally, cytokinin is widely recognized as a central, indispensable regulator in the complex process of natural gall formation across diverse systems (He et al., 2020). In our study, the significant finding of these cytokinins within the gall shells themselves provides strong circumstantial evidence for their active involvement in promoting the sustained cell division and tissue proliferation necessary for gall enlargement (Andreas et al., 2020). Furthermore, the detection of notably high levels of TZR and Zeatin specifically within the *T. acutissimae* larvae is a critical observation. This larval accumulation strongly supports earlier findings and emerging theories suggesting that gall-inducing insects actively produce, sequester, or otherwise manipulate host cytokinin levels as a key strategy in their parasitic interaction (Mapes and Davies, 2001; Yamaguchi et al., 2012). Our study further verifies previous observations that the presence of these specific, active cytokinin forms within the larval body indicates they are not merely passive inhabitants but likely active participants in the hormonal milieu governing gall development. Our experimental results demonstrate significantly higher cytokinin concentrations in the larvae compared to both leaf tissues and gall shells. Building upon previous research findings, we believe that *T. acutissimae* larvae possess the biochemical capability to at least partially synthesize cytokinins themselves. This endogenous production would provide a direct mechanism for the larvae to modulate host plant cytokinin signaling pathways, thereby strategically manipulating host tissue development to sustain and expand the gall environment essential for their own survival and growth (Giron and Glevarec, 2014). The larvae, therefore, appear to be a significant source of cytokinins contributing to the hormonal control of the gall.

The physiological and biochemical responses of plants to gall-inducing attack are not uniform but exhibit significant host species-specificity. Different host plant species, even closely related ones, may exhibit distinct physiological, hormonal, and molecular responses when infested by the same gall-inducing species. A clear example of this phenomenon is demonstrated by two *Eucalyptus* species, which displayed markedly different hormonal profiles and defensive chemical signatures following infestation by the gall wasp *Leptocybe invasa* (Li et al., 2017). This highlights that inherent genetic and physiological differences between hosts fundamentally shape their interaction with a shared parasite. In our study comparing the oak species *Q. variabilis* and *Q. acutissima* infested by the same wasp *T. acutissimae*, the observed differences in plant hormone concentrations among galls, larvae, and surrounding leaves could potentially stem from multiple factors. While genuine interspecies variation is a primary consideration, differences in sampling time or the developmental stage of the galls/larvae at collection represent critical alternative or confounding explanations. Notably, our field observations revealed that galls induced on *Q. acutissima* mature approximately one month later than those on *Q. variabilis*. This temporal disconnect means that samples collected on the same calendar date represent fundamentally different physiological states. Careful stage-matched sampling in future work would be essential to isolate the effect of host species from developmental timing.

### 4.2 Gene expression profiles of galls, larvae, and leaves

Transcriptome analysis uncovered extensive alterations in gene expression associated with auxin and cytokinin signaling pathways during gall formation. By comparing the transcriptomic profiles of galled leaves, ungalled leaves, and galls in *Q. variabilis*, we identified distinct organ specific expression patterns that are closely linked to plant-insect interactions. These findings provide experimental evidence that *T. acutissimae* larvae substantially reprogram *Quercus* host gene expression at both local and systemic levels. Previous transcriptome studies have documented thousands of DEGs activated during gall development (Nabity et al., 2013; Bailey et al., 2015; Hearn et al., 2019; Schultz et al., 2019). Our analysis identified 16,678 DEGs across various metabolic pathways, with many involved in plant hormone biosynthesis and signaling. Auxin-related DEGs were notably altered between gall shells and leaves. While earlier studies reported upregulation of auxin response genes in gall tissues (Tooker and De Moraes, 2011), such as in psyllid galls on *Metrosideros polymorpha* (Bailey et al., 2015) and in aphid-induced galls on *Rhus chinensis* and *R. javanica* (Hirano et al., 2020), our results revealed a contrasting pattern that auxin biosynthesis genes were significantly downregulated in gall tissues, while genes involved in auxin inactivation were upregulated. The downregulation of biosynthetic genes in plant tissues may reflect tissue-specific or developmental temporal regulation, but this expression pattern coincided with the measured IAA levels, which were higher in larvae and leaves than in gall shells. Although studies have reported elevated auxin levels in galls (Mapes and Davies, 2001), our data suggest that *T. acutissimae* larvae, despite containing high levels of IAA, do not significantly increase levels of IAA in galls, echoing previous studies (Tooker and De Moraes, 2011; Yamaguchi et al., 2012). By analogy with the action mechanisms of other phytohormones (e.g., gibberellins), we observed that IAA exhibits similar regulatory patterns in both *T. acutissimae* larvae and their induced galls. This may reflect the fact that the host plant downregulates hormone concentrations to inhibit gall growth, while the gall-inducing insects counteract these efforts (Wang H. et al., 2016). Combining the transcriptomic and hormonal evidence, it can be anticipated that *T. acutissimae* larvae are likely to employ a comparable mechanism for the production and secretion of insect-derived auxin. Following the trend observed for auxin-related genes, most cytokinin synthesis and signaling genes were downregulated in gall tissues compared to leaf tissues, exhibiting a similar pattern. This observed downregulation of cytokinin synthesis and signaling genes may reflect a crucial aspect of the arm race between plants and gall-inducing insects. By modulating hormone concentrations, insects modulate resource allocation of host plants through either indirect effects mediated by physiological defense competence or direct impacts on defense mechanisms (Roitsch and Ehneß, 2000; Balibrea Lara et al., 2004; Berger et al., 2007). ARR17 functions as a rapid-response negative regulator that modulates signal sensitivity through feedback inhibition, whereas ARR14/ARR2 act as transcriptional activators that directly drive the expression of development-related genes. The observed downregulation of ARR2 and ARR14 in our experimental results may represent a defensive strategy—On the other hand, gall-inducing insects counteract the host plant’s efforts by potentially secreting specific substances (including plant growth regulators such as auxins, cytokinins, IAA, and other types of compounds) or triggering molecular pathways that interfere with the plant’s attempt to downregulate hormone concentrations (Gätjens-Boniche, 2019; Takeda et al., 2021). However, our results demonstrate that the concentrations of cytokinins are significantly lower than those of IAA in *T. acutissimae* larvae, suggesting that IAA, rather than CTKs, plays a pivotal role in manipulating hormone levels in galls. This finding contrasts somewhat with the observations reported in previous studies (Yamaguchi et al., 2012), implying that different gall-inducing organisms may employ distinct hormonal mechanisms to modulate the formation of plant galls.

### 4.3 Decrease in jasmonic acid synthesis supports the “nutritional hypothesis”

Plant hormones, particularly jasmonic acid (JA), serve as central orchestrators of the host plant’s innate immune system (Walling, 2000; Tooker et al., 2008). These signaling molecules are pivotal for activating defense responses against biotic stressors, including herbivorous insects, and their complex interplay is intrinsically linked to the initiation and development of insect-induced galls (Tooker et al., 2008). Crucially, gall-inducing insects have evolved sophisticated strategies to subvert these very pathways, actively modulating JA signaling to manipulate host plant physiology, suppress defensive reactions, and redirect resources to create a nutrient-rich, protected environment conducive to larval development (Takei et al., 2015). Our investigation into the interaction between *Quercus* species and the gall wasp *T. acutissimae* provides compelling evidence of this dynamic. Transcriptome analysis revealed several differentially expressed genes (DEGs) associated with JA biosynthesis. Significantly, the majority of these JA-related DEGs exhibited higher expression levels in the undamaged leaves compared to the gall tissues. This transcriptional pattern was robustly corroborated by direct hormone quantification, which substantially decreased JA concentrations in the galls. Collectively, these data strongly indicate that *Quercus* hosts mount a systemic JA-mediated defense response in non-galled tissues as a primary strategy to counter the initial wasp invasion or potentially limit further infestation. The upregulation of JA synthesis and signaling in leaves represents the oak’s attempt to deploy its standard anti-herbivore arsenal. Conversely, within the developing gall itself, the observed downregulation of JA biosynthesis genes points to a starkly different hormonal landscape. Our study identified several key differentially expressed genes (DEGs) associated with jasmonic acid (JA) biosynthesis, including lipoxygenase (LOX) genes. Notably, the majority of these genes exhibited lower expression levels in gall tissues compared to leaf tissues. By actively dampening the host’s JA-dependent defenses specifically at the site of larval feeding, *T. acutissimae* likely creates a more favorable microenvironment by suppressing the synthesis of defensive compounds within gall tissues. This localized suppression of JA production and signaling within the gall structure aligns directly with the ‘nutritional hypothesis’ of gall formation (Price et al., 1987; Takei et al., 2015). This finding aligns with previous research on the aphid *Tetraneura akinire*, which induces significant JA accumulation in infested *Ulmus pumila* leaves (Huang et al., 2016). However, our results demonstrate that JA levels in gall shells were substantially lower than those in leaves, indicating that reduced plant defenses are beneficial to larval development. These findings paint a nuanced picture of defense and counter-defense: The host *Quercus* tree predominantly employs a systemic jasmonic acid (JA)-mediated defense strategy in its foliage to counter the attack by *T. acutissimae*. Meanwhile, within the gall niche, the insect appears to effectively suppress JA-mediated defenses by modulating gene expression. This spatially segregated hormonal reprogramming between leaves and galls underscores the sophisticated manipulation employed by the gall inducer to optimize its parasitic interaction with the host plants.

### 4.4 Gene expression associated with primary metabolism

Our transcriptome analysis revealed that many differentially expressed genes (DEGs) upregulated in gall tissues were associated with carbohydrate metabolism, energy production, translation, amino acid metabolism, protein folding, and lipid metabolism. These changes indicate enhanced biosynthetic activity, increased ribosome biogenesis, and accelerated metabolic fluxes in galls, collectively promoting gall chamber expansion and providing nutrients for larval development (Shi et al., 2019; Korgaonkar et al., 2021).

The impact of galls on host photosynthesis varies across systems. For example, some galls induced by phylloxera and mites reduce photosynthetic activity, whereas others, such as those caused by *Smicronyx madaranus*, have been reported to enhance photosynthetic activity of the shoots of *Cuscuta campestris*, an obligate parasitic plant (Florentine et al., 2005). Most leaf galls, however, are considered carbon sinks, exhibiting lower photosynthetic capacity compared to surrounding tissues (Fay et al., 1993; Larson, 1998). In our study, photosynthesis-related genes were more highly expressed in both galled and non-galled leaves than in gall shells, with only one DEG identified between the two leaf types. The reduced gene expression in gall tissues may result from limited light exposure due to structural shielding. Thus, like other systems, oak cavity galls function as heterotrophic sinks, relying on carbohydrates derived from the host (Chen et al., 2020).

Analysis of primary metabolite-associated DEGs indicated downregulation of starch biosynthesis genes and upregulation of genes related to soluble sugars, amino acids, and fatty acids in gall tissues. These changes suggest a shift from carbon storage to carbon utilization. The upregulation of unsaturated fatty acid biosynthesis genes in gall shells is functionally significant, as these fatty acids are essential for biological membrane functions and play crucial roles in stress resistance mechanisms including drought tolerance, UV protection, and cold acclimation. This enhanced expression of unsaturated fatty acid-related genes, therefore, contributes substantially to both gall development and survival. Upregulation of the sucrose metabolism pathway, including key enzymes such as *fructokinase* and *hexokinase*, indicates enhanced sugar catabolism and energy availability. Martinson et al. (2021) proposed that this phenomenon represents a candidate mechanism for the nutritive tissue of galls functioning as a strong metabolic sink (Egan et al., 2022). This study further demonstrated that altered expression levels of hexokinase genes contribute to gall growth, indicating that the observed downregulation of starch synthesis coupled with enhanced soluble sugar production in galls serves to provide carbohydrate nutrients essential for gall development and expansion, potentially strengthening plant responses to stress (Dai et al., 1999). Notably, the *fructokinase* gene may regulate sucrose breakdown and regulates senescence processes and photosynthetic activity, supporting the role of gall tissues as strong sinks (Egan et al., 2022). Additionally, the upregulation of amino acid transporter genes and elevated levels of soluble sugars and free amino acids in galls further corroborate their nutritional enrichment, consistent with the ‘nutritional hypothesis’.

### 4.5 Synthesis

**Figure 5:**
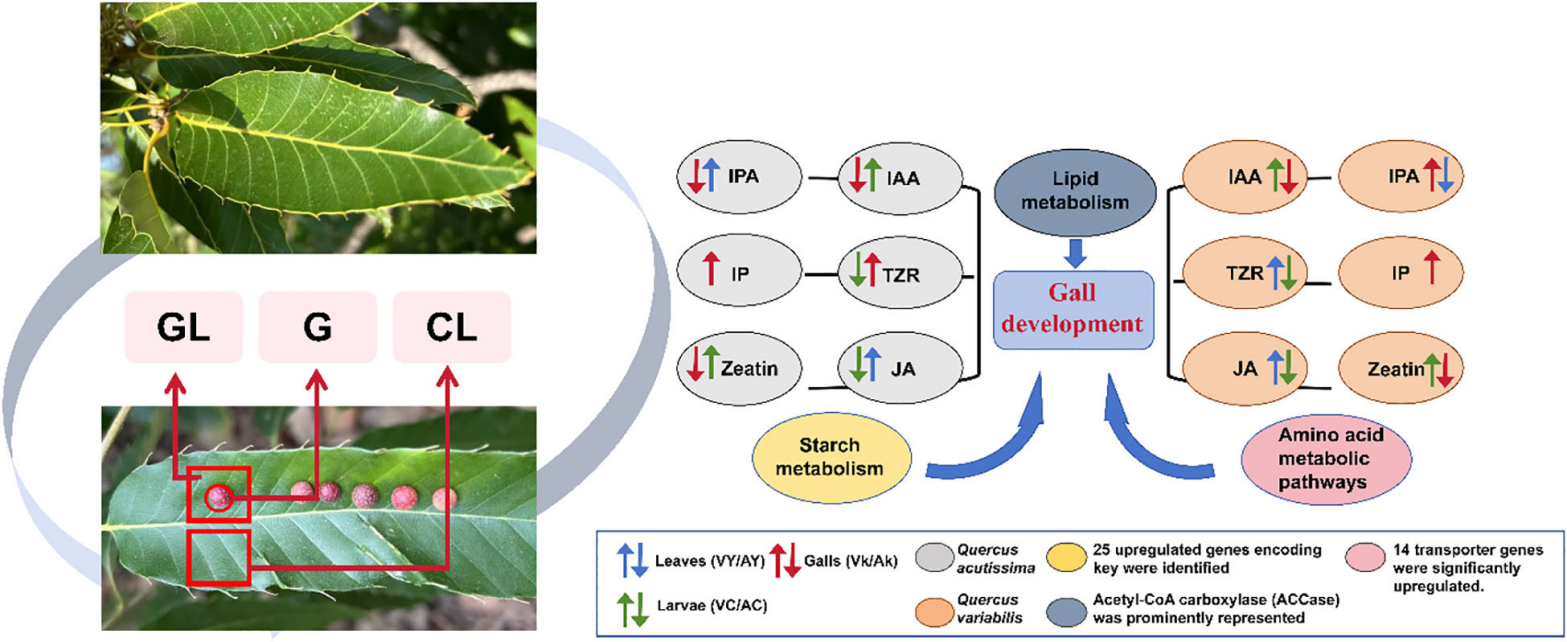
A mechanistic diagram depicting the process by which *Trichagalma acutissimae* induces and manipulates gall formation on the leaves of *Quercus variabilis* and *Quercus acutissima*.

Under normal physiological conditions, *Quercus* species maintain a stable equilibrium between hormone signaling and primary metabolic processes. However, the induction of galls by *T. acutissimae* disrupts this equilibrium, resulting in substantial alterations in hormone concentrations, gene expression profiles, and nutrient distribution. Our integrated analysis, which combines transcriptomic data and hormone quantification, indicates that gall-inducing larvae likely synthesize or accumulate auxins and cytokinins to manipulate host tissue architecture, while simultaneously suppressing JA-mediated defense mechanisms. The reduction in defensive compounds and potentially modified nutrient allocation within the gall may enhance nutrient availability and minimize direct tannin toxicity, thereby providing strong support for the ‘nutritional hypothesis’ of gall development. In summary, we propose three critical processes underlying gall formation: (1) Insect larval-derived phytohormones play a regulatory role in gall development; (2) Extensive alterations in the expression of plant hormone signaling pathway genes occur during gall formation, accompanied by suppression of plant defense mechanisms; and (3) Upregulation of primary metabolism-related genes within galls promotes gall formation and nutrient provisioning (**Figure 5**).

Collectively, our findings may have provided insight into the molecular and physiological underpinnings of gall formation in *Quercus* species, advancing our understanding of plant-insect interactions and serving as a valuable reference for studies on other gall-inducing taxa. However, our study has several limitations that should be acknowledged. First, sampling was conducted at a single developmental stage due to the rapid growth of galls, which limited the ability to perform a comprehensive analysis across the entire gall developmental cycle. Second, the transcriptome analysis was restricted to *Q. variabilis* samples, without incorporating parallel data from *Q. acutissima*. Future studies could adopt a more comprehensive time-series experimental design to systematically investigate the dynamic interactions between gall development and host plants across multiple developmental stages. Such an approach would facilitate a deeper understanding of the molecular mechanisms underlying gall formation.

## Data Availability

The high throughput sequencing raw data are publicly available. This data can be found here: https://www.ncbi.nlm.nih.gov/, accession PRJNA1252022.

## Supplementary material

Supplementary Table 1 Transcriptome assembly quality of samples. G, GL, and CL stand for galls, galled leaves, and control leaves of *Quercus variabilis*, respectively.

Supplementary Table 2 IAA synthesis-related DEGs. G, GL, and CL stand for galls, galled leaves, and control leaves of *Quercus variabilis*, respectively.

Supplementary Table 3 IAA inactivation-related DEGs. G, GL, and CL stand for galls, galled leaves, and control leaves of *Quercus variabilis*, respectively.

Supplementary Table 4 Starch-related DEGs. G, GL, and CL stand for galls, galled leaves, and control leaves of *Quercus variabilis*, respectively.

Supplementary Table 5 CTK-related DEGs. G, GL, and CL stand for galls, galled leaves, and control leaves of *Quercus variabilis*, respectively.

Supplementary Table 6 JA-related DEGs. G, GL, and CL stand for galls, galled leaves, and control leaves of *Quercus variabilis*, respectively.

Supplementary Table 7 Soluble sugar-related DEGs. G, GL, and CL stand for galls, galled leaves, and control leaves of *Quercus variabilis*, respectively.

Supplementary Table 8 Fatty acid-related DEGs. G, GL, and CL stand for galls, galled leaves, and control leaves of *Quercus variabilis*, respectively.

Supplementary Table 9 Amino acid-related DEGs. G, GL, and CL stand for galls, galled leaves, and control leaves of *Quercus variabilis*, respectively.

## References

[B1] Acevedo F. E. Smith P. Peiffer M. Helms A. Felton G. W. (2019). Phytohormones in fall armyworm saliva modulate defense responses in plants. J. Chem. Ecol. 45, 598–609. doi: 10.1007/s10886-019-01079-z, PMID: 31218595

[B2] Andreas P. Kisiala A. Emery R. N. De Clerck-Floate R. Tooker J. F. Price P. W.. (2020). Cytokinins are abundant and widespread among insect species. Plants 9, 208. doi: 10.3390/plants9020208, PMID: 32041320 PMC7076654

[B3] Bailey S. Percy D. M. Hefer C. A. Cronk Q. C. B. (2015). The transcriptional landscape of insect galls: psyllid (Hemiptera) gall formation in Hawaiian Metrosideros polymorpha (Myrtaceae). BMC Genomics 16, 943. doi: 10.1186/s12864-015-2109-9, PMID: 26572921 PMC4647832

[B4] Balibrea Lara M. E. Gonzalez Garcia M.-C. Fatima T. Ehneß R. Lee T. K. Proels R.. (2004). Extracellular invertase is an essential component of cytokinin-mediated delay of senescence. Plant Cell 16, 1276–1287. doi: 10.1105/tpc.018929, PMID: 15100396 PMC423215

[B5] Bartlett L. Connor E. F. (2014). Exogenous phytohormones and the induction of plant galls by insects. Arthropod-Plant Interact. 8, 339–348. doi: 10.1007/s11829-014-9309-0

[B6] Bedetti C. S. Bragança G. P. dos Santos Isaias R. M. (2017). Influence of auxin and phenolic accumulation on the patterns of cell differentiation in distinct gall morphotypes on *Piptadenia* gonoacantha (Fabaceae). Aust. J. Bot. 65, 411–420. doi: 10.1071/BT16257

[B7] Bedetti C. Jorge N. Trigueiro F. Bragança G. Modolo L. Isaias R. (2018). Detection of cytokinins and auxin in plant tissues using histochemistry and immunocytochemistry. Biotech. Histochem. 93, 149–154. doi: 10.1080/10520295.2017.1417640, PMID: 29701111

[B8] Bedetti C. S. Modolo L. V. dos Santos Isaias R. M. (2014). The role of phenolics in the control of auxin in galls of *Piptadenia gonoacantha* (Mart.) MacBr (Fabaceae: Mimosoideae). Biochem. Syst. Ecol. 55, 53–59. doi: 10.1016/j.bse.2014.02.016

[B9] Berger S. Sinha A. K. Roitsch T. (2007). Plant physiology meets phytopathology: plant primary metabolism and plant–pathogen interactions. J. Exp. Bot. 58, 4019–4026. doi: 10.1093/jxb/erm298, PMID: 18182420

[B10] Body M. J. A. Zinkgraf M. S. Whitham T. G. Lin C. H. Schultz J. C. (2019). Heritable phytohormone profiles of poplar genotypes vary in resistance to a galling aphid. MPMI 32, 654–672. doi: 10.1094/MPMI-11-18-0301-R, PMID: 30520677

[B11] Buchfink B. Xie C. Huson D. H. (2015). Fast and sensitive protein alignment using DIAMOND. Nat. Methods 12, 59–60. doi: 10.1038/nmeth.3176, PMID: 25402007

[B12] Chen X. Yang Z. Chen H. Qi Q. Liu J. Wang C.. (2020). A complex nutrient exchange between a gall-forming aphid and its plant host. Front. Plant Sci. 11, 811. doi: 10.3389/fpls.2020.00811, PMID: 32733495 PMC7358401

[B13] Dai N. Schaffer A. Petreikov M. Shahak Y. Giller Y. Ratner K.. (1999). Overexpression of Arabidopsis hexokinase in tomato plants inhibits growth, reduces photosynthesis, and induces rapid senescence. Plant Cell 11, 1253–1266. doi: 10.1105/tpc.11.7.1253, PMID: 10402427 PMC144264

[B14] Davies P. J. (1995). “The Plant Hormones: Their Nature, Occurrence, and Functions,” in Plant Hormones: Physiology, Biochemistry and Molecular Biology. (Netherlands, Dordrecht: Springer), 1–12. doi: 10.1007/978-1-4020-2686-7_1

[B15] Desnitskiy A. G. Chetverikov P. E. Ivanova L. A. Kuzmin I. V. Ozman-Sullivan S. K. Sukhareva S. I. (2023). Molecular aspects of gall formation induced by mites and insects. Life 13, 1347. doi: 10.3390/life13061347, PMID: 37374129 PMC10304984

[B16] Dong Z. Chen Y. (2013). Transcriptomics: advances and approaches. Sci. China Life Sci. 56, 960–967. doi: 10.1007/s11427-013-4557-2, PMID: 24091688

[B17] Dorchin N. Cramer M. D. Hoffmann J. H. (2006). Photosynthesis and sink activity of wasp-induced galls *Inacacia pycnantha* . Ecology 87, 1781–1791. doi: 10.1890/0012-9658(2006)87[1781:PASAOW]2.0.CO;2, PMID: 16922327

[B18] Espírito-Santo M. M. Fernandez G. W. (2007). How many species of gall-inducing insects are there on earth, and where are they? Ann. Entomol. Soc Am. 100, 95–99. doi: 10.1603/0013-8746(2007)100[95:HMSOGI]2.0.CO;2

[B19] Fay P. A. Hartnett D. C. Knapp A. K. (1993). Increased photosynthesis and water potentials in *Silphium integrifolium* galled by cynipid wasps. Oecologia 93, 114–120. doi: 10.1007/BF00321200, PMID: 28313783

[B20] Ferreira B. G. Álvarez R. Bragança G. P. Alvarenga D. R. Pérez-Hidalgo N. Isaias R. M. S. (2019). Feeding and other gall facets: patterns and determinants in gall structure. Bot. Rev. 85, 78–106. doi: 10.1007/s12229-019-09207-w

[B21] Florentine S. K. Raman A. Dhileepan K. (2005). Effects of gall induction by *Epiblema Strenuana* on gas exchange, nutrients, and energetics in *Parthenium Hysterophorus* . Biocontrol 50, 787–801. doi: 10.1007/s10526-004-5525-3

[B22] Gätjens-Boniche O. (2019). The mechanism of plant gall induction by insects: revealing clues, facts, and consequences in a cross-kingdom complex interaction. Rev. Biol. Trop. 67, 1359–1382. doi: 10.15517/rbt.v67i6.33984

[B23] Giron D. Glevarec G. (2014). Cytokinin-induced phenotypes in plant-insect interactions: learning from the bacterial world. J. Chem. Ecol. 40, 826–835. doi: 10.1007/s10886-014-0466-5, PMID: 24944001

[B24] Harris M. O. Pitzschke A. (2020). Plants make galls to accommodate foreigners: some are friends, most are foes. New Phytol. 225, 1852–1872. doi: 10.1111/nph.16340, PMID: 31774564

[B25] He L. Zhang F. Wu X. Hu Y. Dong L. Dewitte W.. (2020). Genome-wide characterization and expression of two-component system genes in cytokinin-regulated gall formation in. Zizania latifolia. Plants 9, 1409. doi: 10.3390/plants9111409, PMID: 33105697 PMC7690396

[B26] Hearn J. Blaxter M. Schönrogge K. Nieves-Aldrey J.-L. Stone G. N. (2019). Genomic dissection of an extended phenotype: Oak galling by a cynipid gall wasp. PloS Genet. 15, e1008398. doi: 10.1371/journal.pgen.1008398, PMID: 31682601 PMC6855507

[B27] Hirano T. Kimura S. Sakamoto T. Okamoto A. Nakayama T. Matsuura T.. (2020). Reprogramming of the developmental program of *Rhus javanica* during initial stage of gall induction by *Schlechtendalia chinensis* . Front. Plant Sci. 11, 471. doi: 10.3389/fpls.2020.00471, PMID: 32499792 PMC7243852

[B28] Huang Z. Li Y. Shi B. (2016). Activity changes of defense enzymes and jasmonic acid content during the galls formation in leaves of *Ulmus pumila* . J. Agric. Univ Hebei 39, 42–48.

[B29] Jia M. Li Q. Hua J. Liu J. Zhou W. Qu B.. (2020). Phytohormones regulate both “fish scale” galls and cones on *Picea koraiensis* . Front. Plant Sci. 11, 580155. doi: 10.3389/fpls.2020.580155, PMID: 33329642 PMC7729011

[B30] Kmieć K. (2025). “Physiological and Biochemical Properties of Aphid Galls,” in Plant Galls: Structure and Functions. (Cham: Springer), 149–163. doi: 10.1007/978-3-031-80064-1_9

[B31] Korgaonkar A. Han C. Lemire A. L. Siwanowicz I. Bennouna D. Kopec R. E.. (2021). A novel family of secreted insect proteins linked to plant gall development. Curr. Biol. 31, 1836–1849.e12. doi: 10.1016/j.cub.2021.01.104, PMID: 33657407 PMC8119383

[B32] Kutsukake M. Uematsu K. Fukatsu T. (2019). Plant manipulation by gall-forming social aphids for waste management. Front. Plant Sci. 10,933. doi: 10.3389/fpls.2019.00933, PMID: 31396247 PMC6664026

[B33] Langmead B. Salzberg S. L. (2012). Fast gapped-read alignment with Bowtie 2. Nat. Methods 9, 357–359. doi: 10.1038/nmeth.1923, PMID: 22388286 PMC3322381

[B34] Larson K. C. (1998). The impact of two gall-forming arthropods on the photosynthetic rates of their hosts. Oecologia 115, 161–166. doi: 10.1007/s004420050503, PMID: 28308447

[B35] Li X. Q. Liu Y. Z. Guo W. F. Solanki M. K. Yang Z. D. Xiang Y.. (2017). The gall wasp *Leptocybe invasa* (Hymenoptera: Eulophidae) stimulates different chemical and phytohormone responses in two *Eucalyptus* varieties that vary in susceptibility to galling. Tree Physiol. 37, 1208–1217. doi: 10.1093/treephys/tpx098, PMID: 28938058

[B36] Lieceng Z. Ming-Shun C. Xiang L. (2011). Changes in phytohormones and fatty acids in wheat and rice seedlings in response to hessian fly (Diptera: Cecidomyiidae) infestation. J. Econ. Entomol. 104, 1384–1392. doi: 10.1603/EC10455, PMID: 21882708

[B37] Mapes C. C. Davies P. J. (2001). Cytokinins in the ball gall of *Solidago altissima* and in the gall forming larvae of *Eurosta solidaginis* . New Phytol. 151, 203–212. doi: 10.1046/j.1469-8137.2001.00158.x, PMID: 33873383

[B38] Martinson E. O. Werren J. H. Egan S. P (2022). Tissue-specific gene expression shows a cynipid wasp repurposes oak host gene networks to create a complex and novel parasite-specific organ. Mol. Ecol. 31, 3228–3240. doi: 10.1111/mec.16159, PMID: 34510608

[B39] Melika G. Pujade-Villar J. Abe Y. Tang ChangTi T. C. Nicholls J. Wachi N.. (2010). Palaearctic oak gallwasps galling oaks (*Quercus*) in the section Cerris: re-appraisal of generic limits, with descriptions of new genera and species (Hymenoptera: Cynipidae: Cynipini). Zootaxa 2470, 1–79. doi: 10.11646/zootaxa.2470.1.1

[B40] Nabity P. D. Haus M. J. Berenbaum M. R. Delucia E. H. (2013). Leaf-galling phylloxera on grapes reprograms host metabolism and morphology. Proc. Natl. Acad. Sci. U. S. A. 110, 16663–16668. doi: 10.1073/pnas.1220219110, PMID: 24067657 PMC3799386

[B41] Ponce G. E. Fuse M. Chan A. Connor E. F. (2021). The localization of phytohormones within the gall-inducing insect. Eurosta solidaginis (*Diptera: Tephritidae*). Arthropod-Plant Interact. 15, 375–385. doi: 10.1007/s11829-021-09817-5, PMID: 34149963 PMC8211092

[B42] Price P. W. Fernandes G. W. Waring G. L. (1987). Adaptive nature of insect galls. Environ. Entomol. 16, 15–24. doi: 10.1093/ee/16.1.15

[B43] Pujade J. Wang J. (2012). A new species of the genus Trichagalma Mayr from China (Hym.: Cynipidae). Orsis 26, 91–101. doi: 10.1093/ee/16.1.15

[B44] Raman A. Madhavan S. Florentine S. Dhileepan K. (2006). Stable-isotope ratio analyses of metabolite mobilization in the shoot galls of *Parthenium hysterophorus* (Asteraceae) induced by *Epiblema strenuana* (Lepidoptera, Tortricidae). Entomol. Exp. Appl. 119, 101–117. doi: 10.1111/j.1570-7458.2006.00403.x

[B45] Raman A. Schafer C. Withers T. M. (2005). Galls and gall-inducing arthropods: an overview of their biology, ecology, and evolution. Biol. Ecol. Evol. Gall-induc. Arthropods 100, 1–33.

[B46] Rezende U. C. Cardoso J. C. F. Hanson P. Oliveira D. C. D. (2021). Gall traits and galling insect survival in a multi-enemy context. Rev. Biol. Trop. 69, 219–301. doi: 10.15517/rbt.v69i1.42826

[B47] Roberts A. Pachter L. (2013). Streaming fragment assignment for real-time analysis of sequencing experiments. Nat. Methods 10, 71–73. doi: 10.1038/nmeth.2251, PMID: 23160280 PMC3880119

[B48] Roberts A. Trapnell C. Donaghey J. Rinn J. L. Pachter L. (2011). Improving RNA-Seq expression estimates by correcting for fragment bias. Genome Biol. 12, 1–14. doi: 10.1186/gb-2011-12-3-r22, PMID: 21410973 PMC3129672

[B49] Roitsch T. Ehneß R. (2000). Regulation of source/sink relations by cytokinins. Plant Growth Regul. 32, 359–367. doi: 10.1023/A:1010781500705

[B50] Roy S. Das A. (2023). Insect-induced foliar galls: a cross-talk among phytohormones for tissue growth and endogenous defense. Khulna Univ. Stud. 8, 36–46. doi: 10.53808/KUS.SI.2023.ICES.A75-ls

[B51] Santos J. Cortez J. A. Fernandes G. (2011). Diversity of gall-inducing insects in the high altitude wetland forests in Pernambuco, Northeastern Brazil. Brazil. Braz. J. Biol. 71, 47–56. doi: 10.1590/S1519-69842011000100008, PMID: 21437398

[B52] Schaefer C. W. Raman A. Withers T. M. Raman A. Schaefer C. W. Withers T. M. (2005). “Galls and gall-inducing arthropods: ecological issues and evolutionary problems,” in Biology, Ecology, and Evolution of Gall-inducing Arthropods. (New Hampshire, USA: Science Publishers, Inc), 761–766.

[B53] Schultz J. C. Edger P. P. Body M. J. A. Appel H. M. (2019). A galling insect activates plant reproductive programs during gall development. Sci. Rep. 9, 1833. doi: 10.1038/s41598-018-38475-6, PMID: 30755671 PMC6372598

[B54] Shi H. Takeda S. Yoza M. Amano T. Ohshima I. Hirano T.. (2019). Comparative transcriptome analysis of galls from four different host plants suggests the molecular mechanism of gall development. PloS One 14, e0223686. doi: 10.1371/journal.pone.0223686, PMID: 31647845 PMC6812778

[B55] Shorthouse J. D. Wool D. Raman A. (2005). Gall-inducing insects – Nature’s most sophisticated herbivores. Basic Appl. Ecol. 6, 407–411. doi: 10.1016/j.baae.2005.07.001

[B56] Suzuki H. Yokokura J. Ito T. Arai R. Yokoyama C. Toshima H.. (2014). Biosynthetic pathway of the phytohormone auxin in insects and screening of its inhibitors. Insect Biochem. Mol. Biol. 53, 66–72. doi: 10.1016/j.ibmb.2014.07.008, PMID: 25111299

[B57] Takeda S. Hirano T. Ohshima I. Sato M. H. (2021). Recent progress regarding the molecular aspects of insect gall formation. Int. J. Mol. Sci. 22, 9424. doi: 10.3390/ijms22179424, PMID: 34502330 PMC8430891

[B58] Takei M. Yoshida S. Kawai T. Hasegawa M. Suzuki Y. (2015). Adaptive significance of gall formation for a gall-inducing aphids on Japanese elm trees. J. Insect Physiol. 72, 43–51. doi: 10.1016/j.jinsphys.2014.11.006, PMID: 25437243

[B59] Tanaka Y. Okada K. Asami T. Suzuki Y. (2013). Phytohormones in Japanese mugwort gall induction by a gall-inducing gall midge. Biosci. Biotechnol. Biochem. 77, 1942–1948. doi: 10.1271/bbb.130406, PMID: 24018692

[B60] Tooker J. F. De Moraes C. M. (2011). Feeding by a gall-inducing caterpillar species alters levels of indole-3-acetic and abscisic acid in Solidago altissima (Asteraceae) stems. Arthropod-Plant Interact. 5, 115–124. doi: 10.1007/s11829-010-9120-5

[B61] Tooker J. F. Helms A. M. (2014). Phytohormone dynamics associated with gall insects, and their potential role in the evolution of the gall-inducing habit. J. Chem. Ecol. 40, 742–753. doi: 10.1007/s10886-014-0457-6, PMID: 25027764

[B62] Tooker J. F. Rohr J. R. Abrahamson W. G. De Moraes C. M. (2008). Gall insects can avoid and alter indirect plant defenses. New Phytol. 178, 657–671. doi: 10.1111/j.1469-8137.2008.02392.x, PMID: 18331430

[B63] Walling L. L. (2000). The myriad plant responses to herbivores. J. Plant Growth Regul. 19, 195–216. doi: 10.1007/s003440000026, PMID: 11038228

[B64] Wang J. Cui J. Wu S.-A. Pujade-Villar J. (2016). Description of the sexual generation of *Trichagalma acutissimae* (Hymenoptera: Cynipidae) and notes on its heterogonic life cycle. J. Asia-Pac. Entomol. 19, 405–413. doi: 10.1016/j.aspen.2016.04.008

[B65] Wang W. Guo W. Tang J. Li X. (2022). Phytohormones in galls on eucalypt trees and in the gall-forming wasp *Leptocybe invasa* (Hymenoptera: Eulophidae). Agric. For. Entomol. 24, 609–617. doi: 10.1111/afe.12525

[B66] Wang H. Liu J. Cui K. Chen H. Yang Z. Wu H.. (2016). Gibberellic acid is selectively downregulated in response to aphid-induced gall formation. Acta Physiol. Plant 38, 1–17. doi: 10.1007/s11738-016-2224-5

[B67] Wang J. Wang X. Zhang Y. Wang J. Xu J. Wu S. (2017). Population dynamics of *Trichagalma acutissimae* (Hymenoptera: Cynipidae) and its natural enemy *Torymus* sp.(Hymenoptera: Torymidae). Chin. J. Biol. Control 33, 326. doi: 10.16409/j.cnki.2095-039x.2017.03.005

[B68] Wang Y. Zhang Y. Li R. Li Y. Cha M. Yi X. (2025). Host plant dependence of the symbiotic microbiome of the gall-inducing wasp. Trichagalma acutissimae. Insects 16, 652. doi: 10.3390/insects16070652, PMID: 40725284 PMC12294959

[B69] Xue S. Zhang Y. Gao S. Lu S. Wang J. Zhang K. (2020). Mitochondrial genome of *Trichagalma acutissimae* (Hymenoptera: Cynipoidea: Cynipidae) and phylogenetic analysis. Mitochondrial DNA Part B 5, 1073–1074. doi: 10.1080/23802359.2020.1721366, PMID: 33366880 PMC7748680

[B70] Yamaguchi H. Tanaka H. Hasegawa M. Tokuda M. Asami T. Suzuki Y. (2012). Phytohormones and willow gall induction by a gall-inducing sawfly. New Phytol. 196, 586–595. doi: 10.1111/j.1469-8137.2012.04264.x, PMID: 22913630

[B71] Zhang C. X. He M. X. Cao Y. Liu J. Gao F. Wang W. B.. (2015). Fungus-insect gall of *Phlebopus portentosus* . Mycologia 107, 12–20. doi: 10.3852/13-267, PMID: 25344264

