# Supplementary material for: Transcriptome and phytohormone analysis reveal mechanism of gall formation by Trichagalma acutissimae larvae on oak leaves
Source: Front Plant Sci. 2025 Sep 18;16:1646230. doi: 10.3389/fpls.2025.1646230 (PMC12488683; doi:10.3389/fpls.2025.1646230)
Supplement: Supplementary Table 1 — Transcriptome assembly quality of samples. G, GL, and CL stand for galls, galled leaves, and control leaves of Quercus variabilis, respectively. [file Supplementaryfile1.docx]

**Table S1** Transcriptome assembly quality of samples. G, GL, and CL stand for galls, galled leaves, and control leaves of *Quercus variabilis*, respectively.

| **Sample** | **Raw**  **Reads**  **(M)** | **Raw Bases**  **(G)** | **Clean Reads**  **(M)** | **Clean Bases**  **(G)** | **Valid Bases**  **(%)** | **Q30**  **(%)** | **GC**  **(%)** |
| --- | --- | --- | --- | --- | --- | --- | --- |
| G1 | 48.18 | 7.23 | 47.73 | 6.95 | 96.21 | 94.31 | 43.42 |
| G2 | 51.11 | 7.67 | 50.66 | 7.39 | 96.43 | 94.37 | 43.67 |
| G3 | 47.61 | 7.14 | 47.15 | 6.87 | 96.13 | 93.96 | 43.93 |
| G4 | 48.31 | 7.25 | 47.90 | 6.96 | 96.09 | 94.01 | 43.90 |
| G5 | 47.42 | 7.11 | 47.01 | 6.85 | 96.30 | 93.93 | 43.75 |
| GL1 | 46.62 | 6.99 | 46.24 | 6.72 | 96.14 | 94.40 | 43.61 |
| GL2 | 48.41 | 7.26 | 48.02 | 7.00 | 96.42 | 94.34 | 43.55 |
| GL3 | 48.96 | 7.34 | 48.48 | 7.05 | 96.03 | 93.78 | 43.73 |
| GL4 | 48.08 | 7.21 | 47.62 | 6.97 | 96.59 | 93.85 | 43.72 |
| GL5 | 50.47 | 7.57 | 50.02 | 7.29 | 96.30 | 93.63 | 43.57 |
| CL1 | 50.54 | 7.58 | 50.09 | 7.29 | 96.19 | 94.36 | 44.06 |
| CL2 | 48.59 | 7.29 | 48.20 | 7.07 | 96.94 | 94.43 | 43.94 |
| CL3 | 49.26 | 7.39 | 48.88 | 7.13 | 96.45 | 94.42 | 43.49 |
| CL4 | 48.42 | 7.26 | 48.00 | 6.98 | 96.14 | 93.92 | 43.61 |
| CL5 | 51.32 | 7.70 | 50.88 | 7.42 | 96.40 | 93.96 | 43.41 |

**Table S2** IAA synthesis-related DEGs. G, GL, and CL stand for galls, galled leaves, and control leaves of *Quercus variabilis*, respectively.

| **Gene**  **name** | **Annotation** | **log_2_FC**  **（G-CL）** | **log_2_FC**  **（G-GL）** | **Regulation**  **G-vs-CL** | **Regulation**  **G-vs-GL** |
| --- | --- | --- | --- | --- | --- |
| QuIAA1 | tryptophan aminotransferase-related protein 2 isoform X1 | -1.8431 | -1.2745 | Down | Down |
| QuIAA2 | probable indole-3-pyruvate monooxygenase YUCCA10 | -6.2674 | - | Down | - |
| QuIAA3 | probable indole-3-pyruvate monooxygenase YUCCA10 isoform X2 | 2.6600 | 3.0939 | Up | Up |
| QuIAA4 | probable indole-3-pyruvate monooxygenase YUCCA10 | -4.7138 | -3.8620 | Down | Down |

**Table S3** IAA inactivation-related DEGs. G, GL, and CL stand for galls, galled leaves, and control leaves of *Quercus variabilis*, respectively.

| **Gene**  **name** | **Annotation** | **log_2_FC**  **（G-CL）** | **log_2_FC**  **（G-GL）** | **Regulation**  **G-vs-CL** | **Regulation**  **G-vs-GL** |
| --- | --- | --- | --- | --- | --- |
| QuIAA5 | 2-oxoglutarate-dependent dioxygenase DAO-like | 2.4916 | 1.6407 | Up | Up |
| QuIAA6 | IAA-amino acid hydrolase ILR1-like | 4.1843 | - | Up | - |
| QuIAA7 | IAA-amino acid hydrolase ILR1-like 1 | 2.0187 | 1.8699 | Up | Up |
| QuIAA8 | IAA-amino acid hydrolase ILR1-like 4 | -4.9203 | -5.2020 | Down | Down |
| QuIAA9 | IAA-amino acid hydrolase ILR1-like 5 | 1.2351 | - | Up | - |
| QuIAA10 | IAA-amino acid hydrolase ILR1-like 5 isoform X1 | 4.1402 | 3.2289 | Up | Up |
| QuIAA11 | IAA-amino acid hydrolase ILR1-like 6 | - | 1.9570 | - | Up |
| QuIAA12 | probable indole-3-acetic acid-amido synthetase GH3.6 | 1.6030 | -3.8473 | Up | Up |
| QuIAA13 | putative indole-3-acetic acid-amido synthetase GH3.9 | 4.1691 | 1.0636 | Up | Up |

**Table S4** CTK-related DEGs. G, GL, and CL stand for galls, galled leaves, and control leaves of *Quercus variabilis*, respectively.

| **Gene**  **name** | **Annotation** | **log_2_FC**  **（G-CL）** | **log_2_FC**  **（G-GL）** | **Regulation**  **G-vs-CL** | **Regulation**  **G-vs-GL** |
| --- | --- | --- | --- | --- | --- |
| QUCTK1 | cytokinin dehydrogenase 5 | -2.5983 | - | Down | - |
| QUCTK2 | histidine-containing phosphotransfer protein 4-like | -8.2146 | -4.8844 | Down | Down |
| QUCTK3 | two-component response regulator ARR14-like | -4.1190 | -4.6788 | Down | Down |
| QUCTK4 | two-component response regulator ARR17-like | 5.8521 | 4.7024 | Up | Up |
| QUCTK5 | two-component response regulator ARR2-like | -3.3923 | -2.6781 | Down | Down |
| QUCTK6 | two-component response regulator ORR26-like | -23.3011 | -22.1335 | Down | Down |
| QUCTK7 | zeatin O-glucosyltransferase-like | 3.1173 | - | Up | - |
| QUCTK8 | UDP-glycosyltransferase 73C6-like | -6.0032 | -4.9338 | Down | Down |
| QUCTK9 | adenylate isopentenyltransferase 5,  chloroplastic-like | -6.0646 | -4.9883 | Down | Down |
| QUCTK10 | adenylate isopentenyltransferase 3,  chloroplastic-like | -1.8769 | - | Down | - |

**Table S5** JA-related DEGs. G, GL, and CL stand for galls, galled leaves, and control leaves of *Quercus variabilis*, respectively.

| **Gene**  **name** | **Annotation** | **log_2_FC**  **（G-CL）** | **log_2_FC**  **（G-GL）** | **Regulation**  **G-vs-CL** | **Regulation**  **G-vs-GL** |
| --- | --- | --- | --- | --- | --- |
| QuJA1 | 12-oxophytodienoate reductase 2-like | -3.5307 | - | Down | - |
| QuJA2 | allene oxide cyclase, chloroplastic-like | - | -2.0668 | - | Down |
| QuJA3 | allene oxide cyclase, chloroplastic-like | 2.2582 | 1.5269 | Up | Up |
| QuJA4 | allene oxide synthase 3-like | -3.9400 | -4.7037 | Down | Down |
| QuJA5 | probable linoleate 9S-lipoxygenase 5 isoform X1 | -1.5666 | - | Down | - |
| QuJA6 | linoleate 13S-lipoxygenase 2-1, chloroplastic-like isoform X6 | -2.9379 | -2.6657 | Down | Down |
| QuJA7 | linoleate 13S-lipoxygenase 2-1, chloroplastic-like isoform X2 | 2.5822 | - | Up | - |
| QuJA8 | linoleate 13S-lipoxygenase 3-1, chloroplastic-like | -1.8292 | -2.4121 | Down | Down |

**Table S6** Starch-related DEGs. G, GL, and CL stand for galls, galled leaves, and control leaves of *Quercus variabilis*, respectively.

| **Gene**  **name** | **Annotation** | **log_2_FC**  **（G-CL）** | **log_2_FC**  **（G-GL）** | **Regulation**  **G-vs-CL** | **Regulation**  **G-vs-GL** |
| --- | --- | --- | --- | --- | --- |
| QuS1 | ADP-glucose pyrophosphorylase small subunit ADPGp-2 | -9.5845 | -9.1678 | Down | Down |
| QuS2 | granule-bound starch synthase 1  chloroplastic/amyloplastic-like | -4.2564 | -3.8756 | Down | Down |
| QuS3 | granule-bound starch synthase 2  chloroplastic/amyloplastic | 1.3343 | - | Up | - |
| QuS4 | probable starch synthase 4, chloroplastic/amyloplastic | -1.6716 | -1.2376 | Down | Down |

**Table S7** Soluble sugar-related DEGs. G, GL, and CL stand for galls, galled leaves, and control leaves of *Quercus variabilis*, respectively.

| **Gene**  **name** | **Annotation** | **log_2_FC**  **（G-CL）** | **log_2_FC**  **（G-GL）** | **Regulation**  **G-vs-CL** | **Regula-ion**  **G-vs-GL** |
| --- | --- | --- | --- | --- | --- |
| QuSS1 | 1,4-alpha-glucan-branching enzyme 1,  chloroplastic/amyloplastic-like isoform X2 | 1.5967 | 1.5888 | Up | Up |
| QuSS2 | 4-alpha-glucanotransferase,  chloroplastic/amyloplastic isoform X1 | 1.4274 | 1.1815 | Up | Up |
| QuSS3 | 4-alpha-glucanotransferase,  chloroplastic/amyloplastic isoform X2 | - | 2.3813 | - | Up |
| QuSS4 | acid beta-fructofuranosidase | 9.0932 | 6.9162 | Up | Up |
| QuSS5 | acid beta-fructofuranosidase 1, vacuolar-like | 4.7442 | 5.4635 | Up | Up |
| QuSS6 | alpha-amylase-like | 3.8135 | 2.7907 | Up | Up |
| QuSS7 | alpha-glucosidase-like | 1.7185 | 1.5286 | Up | Up |
| QuSS8 | beta-amylase isoform X1 | 1.9059 | 2.7021 | Up | Up |
| QuSS9 | beta-glucosidase 18-like isoform X2 | -1.9144 | - | Down | - |
| QuSS10 | beta-glucosidase 40 | -3.5460 | -2.5163 | Down | Down |
| QuSS11 | beta-glucosidase 46-like | -2.8351 | -2.8029 | Down | Down |
| QuSS12 | endoglucanase 24-like | 3.2626 | 3.3768 | Up | Up |
| QuSS13 | endoglucanase 25-like | 3.7708 | 3.5623 | Up | Up |
| QuSS14 | endoglucanase 6 isoform X1 | 3.0718 | 2.8318 | Up | Up |
| QuSS15 | fructokinase-2 | 4.4060 | 4.1164 | Up | Up |
| QuSS16 | glucan endo-1,3-beta-glucosidase 1 | 2.5748 | 2.8334 | Up | Up |
| QuSS17 | glucan endo-1,3-beta-glucosidase 4 | 1.5810 | 1.7365 | Up | Up |
| QuSS18 | glucan endo-1,3-beta-glucosidase 6-like | 3.3482 | 3.2752 | Up | Up |
| QuSS19 | glucose-1-phosphate adenylyltransferase  large subunit 1-like | 3.7657 | 2.2178 | Up | Up |
| QuSS20 | glucose-1-phosphate adenylyltransferase large subunit 3  chloroplastic/amyloplastic | -5.8526 | -5.1816 | Down | Down |
| QuSS21 | glucose-1-phosphate adenylyltransferase large subunit  chloroplastic/amyloplastic-like | 3.4188 | 2.6490 | Up | Up |
| QuSS22 | glucose-6-phosphate isomerase 1, chloroplastic | 1.0961 | 1.3336 | Up | Up |
| QuSS23 | hexokinase-2, chloroplastic | 2.9039 | 2.9707 | Up | Up |
| QuSS24 | hexokinase-3-like | 1.3249 | 1.0671 | Up | Up |
| QuSS25 | isoamylase 3, chloroplastic | - | -1.1845 | - | Down |
| QuSS26 | probable alpha-amylase 2 isoform X1 | -2.2235 | - | Down | - |
| QuSS27 | probable sucrose-phosphate synthase 1 | 1.3808 | - | Up | - |
| QuSS28 | probable sucrose-phosphate synthase 4 | -5.2040 | -4.2924 | Down | Down |
| QuSS29 | sucrose synthase | 6.2948 | 6.0930 | Up | Up |
| QuSS30 | sucrose synthase 3 | 1.6733 | - | Up | - |
| QuSS31 | sucrose synthase-like | 9.3451 | 10.1135 | Up | Up |
| QuSS32 | alpha,alpha-trehalose-phosphate synthase [UDP-forming] 5 | 3.0229 | 2.8955 | Up | Up |
| QuSS33 | alpha,alpha-trehalose-phosphate synthase [udp-forming] 1 | -3.3708 | -3.5650 | Down | Down |

**Table S8** Fatty acid-related DEGs. G, GL, and CL stand for galls, galled leaves, and control leaves of *Quercus variabilis*, respectively.

| **Gene**  **name** | **Annotation** | **log_2_FC**  **（G-CL）** | **log2FC**  **（G-GL）** | **Regulation**  **G-vs-CL** | **Regulation**  **G-vs-GL** |
| --- | --- | --- | --- | --- | --- |
| QuFA1 | 3-oxoacyl-[acyl-carrier-protein] reductase 4-like | 3.8629 | 3.6257 | Up | Up |
| QuFA2 | 3-oxoacyl-[acyl-carrier-protein] synthase 3 A, chloroplastic-like | 1.8979 | 2.1166 | Up | Up |
| QuFA3 | 3-oxoacyl-[acyl-carrier-protein] synthase I, chloroplastic | 2.3644 | 2.5473 | Up | Up |
| QuFA4 | 3-oxoacyl-[acyl-carrier-protein] synthase II, chloroplastic-like | -4.1246 | -3.7794 | Down | Down |
| QuFA5 | acetyl-coenzyme A carboxylase carboxyl transferase subunit alpha,  chloroplastic-like | 1.0535 | 1.2502 | Up | Up |
| QuFA6 | acyl carrier protein 1, chloroplastic-like | 3.4652 | 3.1671 | Up | Up |
| QuFA7 | acyl carrier protein 1, chloroplastic-like isoform X1 | -6.0847 | -4.9914 | Down | Down |
| QuFA8 | acyl carrier protein 1, mitochondrial | 1.6678 | 1.5436 | Up | Up |
| QuFA9 | acyl carrier protein 2, mitochondrial | 1.0713 | 1.2853 | Up | Up |
| QuFA10 | acyl-acyl carrier protein thioesterase ATL3,  chloroplastic-like isoform X1 | 1.7148 | 1.5821 | Up | Up |
| QuFA11 | delta(12)-fatty-acid desaturase FAD2 | 1.4419 | 1.9892 | Up | Up |
| QuFA12 | oleoyl-acyl carrier protein thioesterase,  chloroplastic-like isoform X1 | 2.5542 | 2.5853 | Up | Up |
| QuFA13 | palmitoyl-acyl carrier protein thioesterase, chloroplastic-like | 5.1789 | 5.6174 | Up | Up |
| QuFA14 | stearoyl-[acyl-carrier-protein] 9-desaturase 6, chloroplastic | 7.4889 | 5.1542 | Up | Up |
| QuFA15 | stearoyl-[acyl-carrier-protein] 9-desaturase, chloroplastic | 1.8726 | 2.2193 | Up | Up |
| QuFA16 | stearoyl-[acyl-carrier-protein] 9-desaturase,  chloroplastic isoform X1 | 2.4489 | 2.2216 | Up | Up |

**Table S9** Amino acid-related DEGs. G, GL, and CL stand for galls, galled leaves, and control leaves of *Quercus variabilis*, respectively.

| **Gene**  **name** | **Annotation** | **log_2_FC**  **（G-CL）** | **log_2_FC**  **（G-GL）** | **Regulation**  **G-vs-CL** | **Regulation**  **G-vs-GL** |
| --- | --- | --- | --- | --- | --- |
| QuAA1 | amino acid permease 3-like | -2.5351 | -2.8155 | Down | Down |
| QuAA2 | amino acid permease 6-like | - | 1.9479 | - | Up |
| QuAA3 | amino acid transporter AVT1A | -2.3420 | -1.8875 | Down | Down |
| QuAA4 | amino acid transporter AVT1C-like | 2.1810 | 2.5393 | Up | Up |
| QuAA5 | amino acid transporter AVT1H-like | -2.6841 | - | Down | Down |
| QuAA6 | amino acid transporter AVT1I-like | -3.1924 | -3.2699 | Down | Down |
| QuAA7 | amino acid transporter AVT1I-like isoform X4 | 2.5286 | 3.4359 | Up | Up |
| QuAA8 | amino acid transporter AVT3C-like | - | 1.2437 | - | Up |
| QuAA9 | amino acid transporter AVT6A-like | 3.0874 | 2.1923 | Up | Up |
| QuAA10 | amino acid transporter AVT6A-like isoform X2 | 5.6087 | 2.9616 | Up | Up |
| QuAA11 | amino acid transporter AVT6C-like | 3.8911 | 3.3166 | Up | Up |
| QuAA12 | amino acid transporter AVT6E-like | - | 1.2766 | - | Up |
| QuAA13 | cationic amino acid transporter 2, vacuolar-like | - | 1.6634 | - | Up |
| QuAA14 | cationic amino acid transporter 4, vacuolar-like isoform X1 | - | 1.1063 | - | Up |
| QuAA15 | cationic amino acid transporter 7, chloroplastic-like | 3.9797 | 2.8512 | Up | Up |
| QuAA16 | LOW QUALITY PROTEIN: cationic amino acid transporter 8, vacuolar-like | - | 1.6848 | - | Up |
| QuAA17 | probable amino acid permease 7 | 2.7666 | 2.2833 | Up | Up |
| QuAA18 | probable vacuolar amino acid transporter YPQ3 | - | 1.5818 | - | Up |
